# In Vitro Evaluation of Olorofim and Amphotericin B Combination Therapy Against *Talaromyces marneffei*

**DOI:** 10.3390/jof12060441

**Published:** 2026-06-17

**Authors:** Le Hoang Ngoc Lan, Heera Natesan Sambath, Lottie Brown, Nguyen Thi Mai Thu, Shawin Vitsupakorn, Phan Thi Ha My, Dang Hoang Khanh, Nguyen Thi Thu Hoai, Ngo Thi Hoa, Thuy Le

**Affiliations:** 1 International University, Ho Chi Minh City 700000, Vietnam; lehoangngoclanbiotech@gmail.com (L.H.N.L.); ntthoai@hcmiu.edu.vn (N.T.T.H.); 2 Vietnam National University, Ho Chi Minh City 720325, Vietnam; 3 Tropical Medicine Research Center for Talaromycosis, Biomedical Research and Diagnostics Center, Pham Ngoc Thach University of Medicine, Ho Chi Minh City 700000, Vietnam; lottiebrown1995@gmail.com (L.B.); thu.t.nguyen@duke.edu (N.T.M.T.); mypth@pnt.edu.vn (P.T.H.M.); khanhdh@pnt.edu.vn (D.H.K.); hoant@pnt.edu.vn (N.T.H.); 4 Division of Infectious Diseases and International Health, Duke University School of Medicine, Durham, NC 27710, USA; 5 Department of Antimicrobial Pharmacodynamics and Therapeutics, University of Liverpool, Liverpool L69 7ZB, UK; 6 The Johns Hopkins University School of Medicine, Baltimore, MD 21205, USA; svitsup1@jh.edu; 7 Oxford University Clinical Research Unit, Ho Chi Minh City 70000, Vietnam; 8 Nuffield Department of Medicine, University of Oxford, Oxford OX1 2JD, UK

**Keywords:** *Talaromyces marneffei*, talaromycosis, amphotericin B, olorofim, drug combination, synergy

## Abstract

The dimorphic fungus *Talaromyces marneffei* causes talaromycosis, a life-threatening fungal disease with limited treatment options. Olorofim, a first-in-class orotomide antifungal that targets pyrimidine synthesis essential for fungal growth, has low minimum inhibitory concentration (MIC) against *T. marneffei* and clinical efficacy against other invasive fungal diseases. Here, we tested the hypothesis that olorofim synergistically enhances amphotericin B (AmB), a potent membrane-targeting antifungal, against *T. marneffei* in 55 clinical isolates using a validated colorimetric checkerboard assay. The MIC was defined as the lowest drug concentration inhibiting ≥ 95% of fungal growth. Drug interactions were assessed using the fractional inhibitory concentration index (FICI), which defines ≤0.5 as synergy, 0.5 < FICI ≤ 4.0 as indifference, and FICI > 4 as antagonism. We found that interactions between AmB and olorofim were indifferent across all 55 isolates (0.5 < FICI ≤ 1.03). Time-kill assays showed an expected concentration-dependent fungicidal activity for AmB, but a concentration-independent fungistatic activity for olorofim against *T. marneffei*. Combinations of AmB and olorofim were also indifferent in time-kill experiments. Although synergy was not observed, and olorofim is unlikely to enhance AmB induction therapy, olorofim may have a role in the consolidation and maintenance therapy of talaromycosis.

## 1. Introduction

### 1.1. Therapeutic Gaps in Talaromycosis

The dimorphic fungus *Talaromyces marneffei* causes talaromycosis, an invasive fungal disease endemic to South and Southeast Asia. Over the last three decades, talaromycosis has emerged as a leading cause of death in people with advanced HIV disease and is increasingly reported among individuals with other immunocompromising conditions and travelers returning from outside of endemic regions [1,2,3]. Mortality remains unacceptably high, ranging between 15% and 50% despite antifungal therapy [4,5]. International treatment guidelines recommend induction therapy with intravenous amphotericin B (AmB) for 10 to 14 days, followed by consolidation and maintenance therapy with oral itraconazole for at least 12 months, based on a single randomized controlled trial [6,7]. AmB deoxycholate is highly fungicidal against *T. marneffei* and effective as induction therapy but causes significant toxicity due to infusion-related reactions, nephrotoxicity, and electrolyte abnormality. As a large, poorly soluble, and highly protein-bound molecule, AmB deoxycholate has variable tissue penetration with limited distribution into certain sites, such as the central nervous system (CNS) [8,9,10]. Itraconazole is better tolerated but has reduced fungicidal activity, poor oral bioavailability, and non-linear pharmacokinetics [11]. Itraconazole is metabolized by the cytochrome P450 3A4 (CYP3A4) enzyme system, resulting in numerous drug interactions, most notably with the anti-tuberculosis drug rifampicin and the antiretroviral drugs efavirenz and ritonavir/cobicistat [11,12]. Therapeutic drug monitoring (TDM) is recommended but is rarely available in resource-limited settings [11]. The limited treatment options contributed to the inclusion of talaromycosis in the 2022 World Health Organization (WHO) Fungal Priority Pathogen List [13], highlighting an urgent need for safer and more effective treatments for this neglected fungal disease.

### 1.2. Olorofim as a Potential Therapeutic Option for Talaromycosis

**Figure 1:**
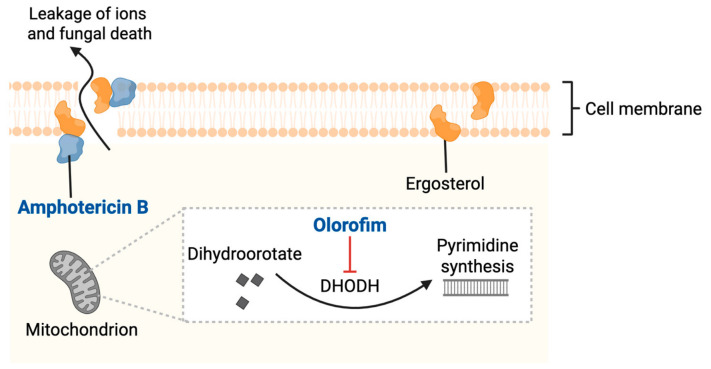
Mechanisms of action of amphotericin B and olorofim. Amphotericin B binds to ergosterol in the fungal cell membrane, leading to the disruption of membrane integrity and the leakage of intracellular ions, ultimately causing fungal cell death. Olorofim inhibits fungal dihydroorotate dehydrogenase (DHODH), a key enzyme in the de novo biosynthesis of pyrimidine, the building blocks for fungal DNA and RNA synthesis.

Olorofim is a first-in-class orotomide antifungal that inhibits dihydroorotate dehydrogenase (DHODH), a key enzyme in the fungal pyrimidine biosynthesis pathway essential for cell wall synthesis and fungal growth (Figure 1) [14]. Olorofim has several favorable pharmacological properties including high oral bioavailability (~68%) and a half-life of 20 to 30 h, allowing for once daily oral dosing [15]. Olorofim exhibits extensive tissue distribution, achieving high tissue-to-plasma ratios (>3:1) in the lungs, liver, and kidneys and near-equivalent levels in the CNS, supporting its potential for fungal infections involving the reticuloendothelial system such as talaromycosis [16,17]. Although olorofim is metabolized by CYP3A4 and other CYP450 enzymes, it has a lower propensity for drug–drug interactions than itraconazole, with only weak interactions expected with CYP3A4 inducers such as rifampicin [18].

Several in vitro and in vivo studies have reported excellent antifungal activity of olorofim against difficult-to-treat molds, including azole-resistant *Aspergillus* spp., *Scedosporium* spp., *Lomentospora prolificans*, *Microascus* spp., *Scopulariopsis* spp., as well as the dimorphic fungi *Histoplasma capsulatum* and *Coccidioides* spp. [19,20,21,22,23,24]. The antifungal effect of olorofim is generally classified as fungistatic in vitro, although some studies show fungicidal activity at higher exposure [25]. Olorofim lacks activity against *Candida* spp., *Cryptococcus* spp., and other yeasts due to variations in the DHODH enzyme [14,22]. A major advantage of olorofim is its favorable safety profile. Gastrointestinal disturbance and transaminitis are the most common adverse events reported with the oral formulation (both occurring in around 10% of people), but these are usually mild and self-limiting [22]. The most serious adverse event reported is drug-induced liver injury, observed in up to 8% of people but only necessitating dose reduction or discontinuation in around 2–3% of cases [18,22]. We and others have shown that olorofim exhibits potent in vitro activity against *T. marneffei*, with minimum inhibitory concentration (MIC) values ranging from 0.008 to 0.002 µg/mL [26,27,28], well below the trough concentrations achieved with standard oral dosing (90 mg, C_min_ > 0.2 µg/mL) [18]. Together, these data provide a strong rationale for further evaluation of olorofim as a treatment for talaromycosis.

### 1.3. Combination Antifungal Therapy Strategy and Rationale for Investigating AmB and Olorofim

Combination antifungal therapy strategy is increasingly used to combat severe and refractory invasive fungal diseases, especially during the early phase of treatment when susceptibility profiles are unknown and to limit the emergence of antifungal resistance in recalcitrant infections [29,30]. Combining agents from different classes with distinct mechanisms of action has been shown to enhance antifungal effects and improve patient outcomes in cryptococcal meningitis (AmB-flucytosine), aspergillosis (voriconazole-anidulafungin), and candidiasis (AmB-flucytosine and AmB-fluconazole for fluconazole-sensitive strains) [29,30,31,32]. We have recently demonstrated in vitro synergy between AmB and flucytosine against *T. marneffei*, providing the proof-of-concept for an ongoing phase 3 Liposomal Amphotericin B and Flucytosine Antifungal Strategy for Talaromycosis (LAmB-FAST) clinical trial (NCT06525389) [33]. Given that olorofim targets the same pyrimidine biosynthesis pathway as flucytosine, we hypothesized there would be in vitro synergy between AmB and olorofim, resulting in enhanced fungal killing against *T. marneffei*.

## 2. Materials and Methods

### 2.1. Selection of Talaromyces marneffei Isolates

Fifty-five *T. marneffei* clinical isolates were randomly selected from a biorepository of 336 clinical isolates collected in the Itraconazole *versus* Amphotericin B for Penicilliosis (IVAP) clinical trial [7]. All 55 isolates came from the blood of patients with advanced HIV (median CD4 count = 11 cells/mm^3^, range: 2–373) who had disseminated talaromycosis involving multiple organ systems, including the lungs, liver, spleen, lymph nodes, skin, bone marrow, and blood stream. All available isolates in the IVAP trial underwent whole genome sequencing, which revealed two phylogenetically and geographically distinct clades of *T. marneffei* in Vietnam [34]. In the present study, 23 isolates came from northern Vietnam and 32 came from southern Vietnam; thus, the sample size of 55 is a good representation of patients from both geographic regions of Vietnam and of isolates from both phylogenetic clades [33], while it is comparable to those used in previous in vitro evaluations of antifungal drugs [35].

### 2.2. Isolate Preparation

*T. marneffei* isolates were stored at −80 °C, revived on Sabouraud dextrose agar (SDA) plates, and grown in the yeast form at 37 °C. After 3–7 days, a single colony was sub-cultured on a new SDA plate to achieve purity. Isolates were sub-cultured once more on SDA plates 3 days before each experiment to ensure uniform growth.

### 2.3. Preparation of Antifungal Drugs

Master stocks of AmB and olorofim were prepared according to the Clinical and Laboratory Standard Institute (CLSI) guidelines [36]. Powders of AmB (Sigma-Aldrich, St. Louis, MO, USA) and olorofim (F2G, Princeton, NJ, USA) were dissolved in dimethyl sulfoxide (DMSO; Sigma-Aldrich, St. Louis, MO, USA). The master stock solutions of AmB (5 mg/mL) and olorofim (1 mg/mL) were aliquoted and stored at −20 °C for up to 6 months until use.

### 2.4. Determination of AmB and Olorofim Combination Effect by Checkerboard Assay

**Figure 2:**
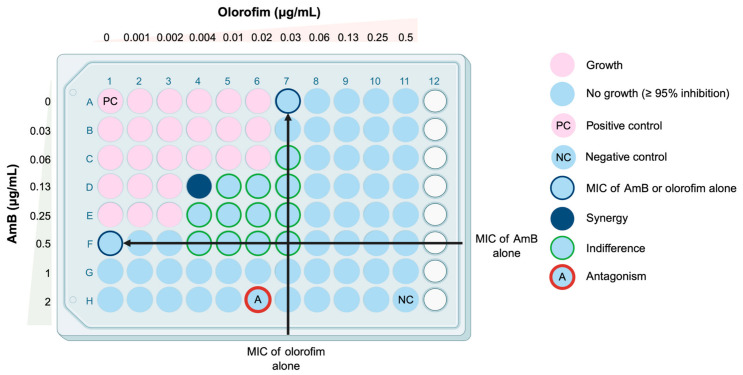
Checkerboard assay method demonstrating interactions between amphotericin B (AmB) and olorofim against *Talaromyces marneffei.* The positive control well (“PC”, position A1) contained no drug and allowed for uninhibited fungal growth. The negative control well (“NC”, position H11) contained no inoculum. A well showing synergy (FICI ≤ 0.5) is highlighted in dark blue (position D4). Wells showing indifference (0.5 < FICI ≤ 4) are highlighted with green outline. A well showing antagonism (FICI > 4) is highlighted in light blue with a red outline (position H6). Wells illustrating the MIC of each drug alone are highlighted in light blue with dark blue outlines (positions A7 and F1). Abbreviations: AmB, amphotericin B; FICI, fractional inhibitory concentration index; MIC, minimum inhibitory concentration.

The checkerboard assay was conducted using our previously validated CLSI-based colorimetric antifungal susceptibility assay [35]. Figure 2 illustrates the checkerboard assay design, which enables the simultaneous determination of MIC for each antifungal tested alone and in combination. Master stock solutions of AmB and olorofim were subjected to twofold serial dilution in Roswell Park Memorial Institute (RPMI) 1640 medium buffered with 3-(N-morpholino) propane sulfonic acid (MOPS) (RPMI-MOPS) (Sigma-Aldrich, St. Louis, MO, USA) and adjusted to pH 7.0. We prepared drug plates with at least one to two dilutions above and below the expected MIC range for each drug. In addition to accounting for the different MIC ranges of AmB and olorofim, we needed to cover MIC ranges that capture potential interactions between AmB and olorofim [27,28,33]. In the 96-well plates, rows contained twofold dilutions of AmB, and columns contained twofold dilutions of olorofim (Figure 2). Positive control wells consisted of RPMI-MOPS plus inoculum and allowed for uninhibited fungal growth. Negative control wells contained only RPMI-MOPS. The prepared drug plates were sealed and stored at −20 °C for up to one month.

On the day of inoculation, *T. marneffei* stock suspension was prepared at an optical density (OD; at 600 nm wavelength) of 0.52–0.58, corresponding to 1–5 × 10^6^ colony-forming units per mL (CFUs/mL). An OD reading at 600 nm was used to estimate inoculum turbidity, as this wavelength is routinely used to measure the cell concentration of *Saccharomyces*, which has a cell size comparable to that of *T. marneffei* (5–10 µm) [37]. In addition, the handheld spectrophotometer used in the study operates only at 600 nm, is cost-effective, and enables convenient use within biosafety cabinets, thereby reducing biosafety risks during inoculum preparation. The inoculum was further diluted with RPMI-MOPS to a final concentration of 1–5 × 10^3^ CFUs/mL. This working inoculum was added to all wells, excluding the negative controls. The plates were sealed and incubated at 37 °C. After 24 h, alamarBlue, a resazurin-based dye that changes from blue to fluorescent pink in proportion to cell metabolism, was added to all the wells. The plates were returned to the incubator for a further 48 h, after which the plates were read by two methods: (i) qualitative visual assessment of color change of alamarBlue and (ii) quantitative assessment of fluorescence intensity (FI; excitation 570 nm, emission 590 nm) using a multimode plate reader (Perkin Elmer EnVision^®^ 2105, Waltham, MA, USA). The growth reduction in each well was calculated using Equation (1).

(1) $$\mathrm{Growth}\;\mathrm{reduction}\;(\%)=\left(1-\frac{{FI}_{sample}-{FI}_{negative\;control}}{{FI}_{positive\;control}-{FI}_{negative\;control}}\right)\times 100$$

While prior studies use 100% growth inhibition as MIC endpoints for AmB and olorofim [27,28,38], complete growth suppression of 100% is technically problematic because real laboratory measurements are never perfectly binary. We used a spectrophotometric threshold of ≥95% inhibition. This adjustment accounts for background noise, biological variability, and machine signal fluctuations near complete inhibition. The use of a 95% endpoint provides the most accurate and practical approximation near complete inhibition, in line with previous susceptibility studies [33,35]. The combination effect of AmB and olorofim was characterized using the fractional inhibitory concentration (FIC), and interactions were measured using the FIC index (FICI), calculated according to Equations (2) and (3), respectively.

(2) $${\mathrm{FIC}}_{\mathrm{drug}}=\frac{MIC\;of\;drug\;in\;combination}{MIC\;of\;drug\;alone}$$

FIC index (FICI) = FIC _AmB_ + FIC _olorofim_ (3)

Based on the FICI, interactions between AmB and olorofim were classified as synergy (FICI ≤ 0.5), indifference (0.5 < FICI ≤ 4), and antagonism (FICI > 4) [39,40].

### 2.5. Determination of Dynamic Effect of AmB and Olorofim and Their Combination in Time-Kill Assay

Time-kill assays were performed to evaluate the dynamic antifungal activity of AmB and olorofim over time, alone and in combination, against a randomly selected isolate, 11CN-03-002. Time-kill assays were performed as previously described, with minor modifications [41]. Briefly, 10 mL tubes containing *T. marneffei* suspensions (10^5^ cells/mL) in RPMI-MOPS and various concentrations of AmB and olorofim were incubated at 37 °C at 190 rpm. To assess concentration-dependent effects, AmB was tested at 0.25×, 0.5×, and 1× the MIC, and olorofim at 1×, 20×, and 50× the MIC, based on the results of the checkerboard assay. A drug-free control tube was included to measure uninhibited fungal growth. For interaction studies, a sub-MIC concentration of AmB (at 0.25× the MIC) was tested with increasing concentrations of olorofim (1×, 20×, 50× the MIC) to detect potential additive or synergistic effects that may be obscured at fully inhibitory concentrations [33]. At 0, 24, 48, 72, and 96 h, 0.1 mL were collected from each tube, serially diluted based on the expected CFUs/mL per testing condition, and incubated on SDA plates. After incubation at 37 °C for 3–5 days, colonies were counted, and CFUs/mL were calculated at each time point.

Fungicidal activity was defined as ≥3 log_10_ CFUs/mL (≥99.9%) reduction from the starting inoculum, while fungistatic activity was defined as <3 log_10_ (<99.9%) reduction [41]. Synergy in the time-kill assay was defined as a reduction of ≥2 log_10_ CFUs/mL in combination compared to the most active agent alone. Antagonism was defined as a ≥2 log_10_ CFUs/mL increase in combination compared to the most active agent alone. A <2 log_10_ decrease in CFUs/mL was classified as indifference [41].

### 2.6. Quality Control

Antifungal drug stock solutions were standardized based on the CLSI guidelines and the published MICs. MICs were first determined against the reference strains *Candida krusei* ATCC 6258 for AmB, and a clinical isolate of *Aspergillus fumigatus* for olorofim [42], in accordance with CLSI M27-A3 for yeasts [36] and M38-A2 for molds [43]. We used two internal reference isolates of *T. marneffei* (Tm North: 11CN-20-091 and Tm South: 11CN-03-130) that have been whole-genome sequenced and clinically and geographically characterized [44] to monitor assay performance and reproducibility. On each day of inoculation, the final *T. marneffei* inoculum was grown on SDA plates at 37 °C for 5 days to check the viability of the inoculum used. An acceptable CFU count was 10–60 colonies, corresponding to 1–6 × 10^3^ cells/mL.

### 2.7. Statistical Analysis

Across the 55 isolates, the modes and geometric means (GMs) of MICs with their 95% confident intervals (95% CIs) were calculated. For FICs and FICI values, arithmetic means ± standard deviations were calculated. Paired t-tests were used to compare the mean MIC differences among the groups: AmB alone vs. AmB in combination, and olorofim alone vs. olorofim in combination. As MICs were obtained from twofold serial dilutions, the data were log_2_-transformed for the analysis and visualization of fold changes. All statistical tests were performed in RStudio (Version 4.5.2; Posit, Boston, MA, USA). Graphs and time-kill plots (log_10_ CFUs/mL over time) were generated using GraphPad Prism (Version 10.1.1; GraphPad Software, Boston, MA, USA), and illustrations were created using BioRender (Toronto, ON, Canada).

The clinical isolates and raw data from the checkerboard and time-kill assays are included in the Supplementary Materials.

### 2.8. Ethics Statement

This study utilized *T. marneffei* clinical isolates obtained from participants of the multi-center IVAP randomized controlled trial [7], which was approved by all five study sites in Vietnam, the Oxford Tropical Research Ethical Committee (OxTREC) in the UK, and the Vietnam Ministry of Health (clinical trial registration: ISRCTN59144167, MoH approval: 3300/QD-BYT). All participants provided written informed consent for specimens to be stored in the IVAP biobank at Duke University and used in this research (Duke IRB protocol: Pro00101915).

## 3. Results

### 3.1. Interaction Between AmB and Olorofim in Checkerboard Assay

**Figure 3:**
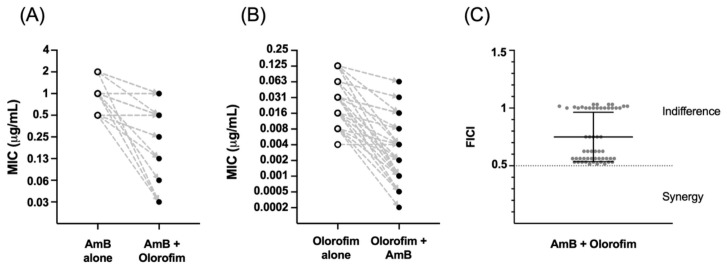
Minimum inhibitory concentrations of amphotericin B (AmB) and olorofim when tested alone and in combination, and the distribution of fractional inhibitory concentration indices in 55 *Talaromyces marneffei* clinical isolates. (**A**) Minimum inhibitory concentrations of AmB when tested alone and in combination. Dashed lines connect results from the same isolate; (**B**) Minimum inhibitory concentrations of olorofim when tested alone and in combination. Dashed lines connect isolates from the same isolate; (**C**) The distribution of fractional inhibitory concentration indices in 55 *T. marneffei* clinical isolates. The dotted line indicates the synergy threshold (FICI < 0.5). and the solid horizontal line indicates the median FICI value. Abbreviations: AmB, amphotericin B; FICI, fractional inhibitory concentration index; MIC, minimum inhibitory concentration.

Table 1 presents the MIC_95_ values for AmB and olorofim tested alone and in combination, together with the corresponding FIC and FICI values for 55 clinical isolates. The MIC_95_ range for AmB was 0.5–2 µg/mL (GM 0.95 µg/mL), and for olorofim, it was 0.004–0.125 µg/mL (GM 0.03 µg/mL). Figure 3 illustrates the changes in MICs and the distribution of FICI. When combined, AmB reduced the MIC_95_ of olorofim by eightfold on average (by 3 dilutions) (Figure 3B), but olorofim only reduced the MIC_95_ of AmB by twofold (1 dilution) (Figure 3A) (*p* < 0.0001 for both groups by paired t-test). (Figure 3C) All isolates demonstrated indifference (0.5 < FICI ≤ 1.03) according to standard FICI criteria. No antagonism was observed in any isolate.

### 3.2. Antifungal Dynamics of AmB and Olorofim Alone over Time in Time-Kill Assay

**Figure 4:**
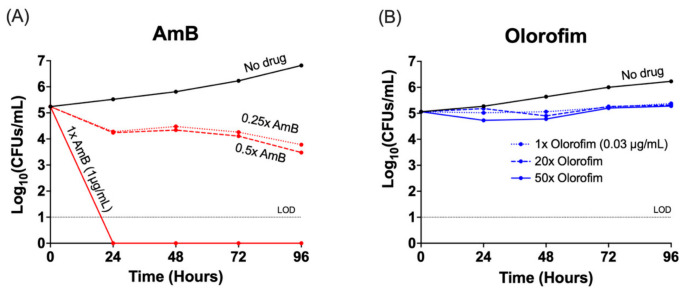
Dynamic antifungal activity of amphotericin B (AmB) and olorofim alone against *Talaromyces marneffei* in time-kill assays. (**A**) AmB exhibited concentration-dependent fungicidal activity, with 5-log reductions from the starting inoculum at 1× the MIC by 96 h. (**B**) Olorofim exhibited <1 log reduction compared to the starting inoculum across 1×, 20×, and 50× the MIC over 96 h, indicating concentration-independent fungistatic activity against *T*. *marneffei.* Abbreviations: AmB, amphotericin B; LOD, limit of detection; CFU, colony-forming unit.

Figure 4 shows the results of the time-kill assay performed on isolate 11CN-03-002 for AmB (Figure 4A) and for olorofim (Figure 4B). AmB exhibited an expected concentration-dependent fungicidal activity against *T. marneffei,* achieving a 5 log_10_ reduction in CFU counts at the MIC of 1 µg/mL. In contrast, olorofim exhibited concentration-independent fungistatic activity across 1×, 20×, and 50× the MIC, with no significant difference in CFU reduction (≤1 log_10_) between concentrations.

### 3.3. Indifference Between AmB and Olorofim Combination in Time-Kill Assay

**Figure 5:**
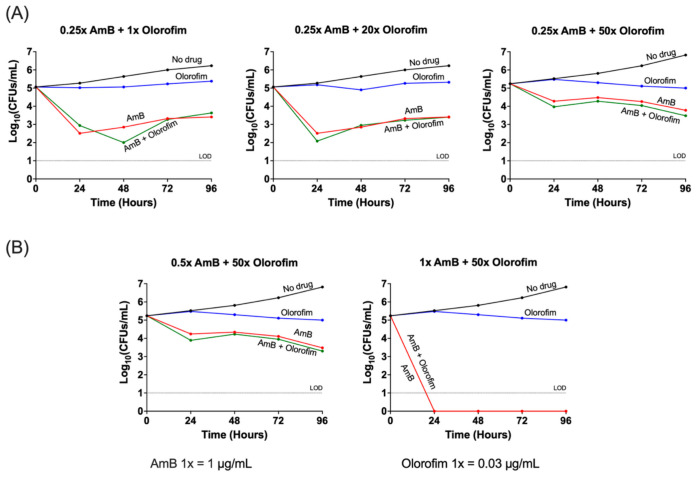
Dynamic antifungal activity of amphotericin B (AmB) and olorofim alone and in combination against *Talaromyces marneffei* in time-kill assays. (**A**) Olorofim did not enhance the antifungal activity of AmB at the sub-MIC concentration of 0.25× the MIC across multiple increasing concentrations of olorofim (1×, 20×, and 50× the MIC). (**B**) Olorofim did not enhance the antifungal activity of AmB at higher AmB concentrations of 0.5× and 1× the MIC and at the highest concentration of olorofim at 50x the MIC, yielding a <1 log reduction in CFUs/mL compared to AmB alone, confirming indifference interaction. No antagonism was observed.Abbreviations: AmB, amphotericin B; LOD, limit of detection; CFU, colony forming unit.

Due to the rapid fungicidal activity of AmB at the MIC (Figure 4A), sub-MIC concentrations (0.25× and 0.5× the MIC) were used in combination experiments to allow for the detection of any additional antifungal effect of olorofim (at 1×, 20×, and 50× the MIC). Across all testing conditions, interaction was indifferent, with no combinations showing a greater than 1 log_10_CFUs/mL reduction in growth compared to AmB alone over 96 h. As shown in Figure 5, the combination of AmB and olorofim closely followed the activity of AmB alone across all concentrations tested. No antagonism was observed.

## 4. Discussion

### 4.1. Summary of Major Findings

We observed indifference between AmB and olorofim across all 55 *T. marneffei* clinical isolates, in both checkerboard and time-kill assays. Although the addition of AmB to olorofim resulted in an eightfold reduction in the MIC of olorofim, this effect was not bidirectional. Olorofim did not significantly enhance the antifungal activity of AmB, either at increasing concentrations of olorofim up to 50× the MIC or at sub-MIC concentrations of AmB (0.25× and 0.5× MIC). Antagonism was not observed either, suggesting that there may be no meaningful interaction between these two agents. Despite the very low MIC (GM 0.03 µg/mL, range 0.004–0.125), in line with previous reports [26,27], olorofim exhibited a fungistatic rather than fungicidal effect in time-kill experiments, having only 1 log_10_ reduction in CFUs/mL compared with the no drug control across a broad range of concentrations over 96 h. AmB, however, showed an expected rapid, concentration-dependent fungicidal effect, consistent with clinical observations. Our findings suggest that olorofim is unlikely to further enhance the robust fungicidal activity of AmB during induction therapy. However, given its significantly lower MICs compared to other azoles against *T. marneffei* isolates, olorofim may still have a role during the consolidation and maintenance phases of talaromycosis treatment and should be further evaluated in in vivo models and clinical studies.

### 4.2. Postulated Mechanism Underlying Interaction

Our hypothesis was built on prior observations of synergy between AmB and flucytosine [33,45], given that olorofim targets the same pyrimidine biosynthesis pathway as flucytosine. The lack of evidence of in vitro synergy may be explained by differences in the rates of antifungal action between the agents. AmB exerts rapid fungicidal activity through ergosterol binding and cell membrane disruption [46]. In contrast, olorofim acts more slowly by inhibiting DHODH, depleting pyrimidine pools, and eventually halting fungal growth [14]. This mismatch in fungal killing kinetics may limit our ability to detect synergy in vitro, with the rapid fungicidal activity of AmB dominating the overall antifungal response and masking the slower, metabolism-based effects of olorofim. In vitro assays also do not capture the complexity of drug distribution across tissue compartments. AmB has limited penetration into certain sites, whereas olorofim demonstrates extensive tissue distribution [8,16]. As a result, potential complementary action between these agents arising from differential tissue distribution in vivo may not be captured in vitro.

### 4.3. Bench to Bedside: The Efficacy of Olorofim Against Other Invasive Fungal Diseases

In the search for new antifungal combinations, it is recognized that in vitro activity does not always translate into in vivo or clinical efficacy [29,47,48]. In our study, AmB and olorofim demonstrated no meaningful interaction. Previous studies of olorofim with AmB or azoles have also shown indifference against most *Aspergillus* spp., with occasional antagonism reported, particularly with *A. niger* [25,38]. However, these findings do not negate the antifungal efficacy of olorofim. In a murine model of invasive pulmonary aspergillosis, olorofim achieved 10-day survival rates ranging from 63 to 88% across three *Aspergillus* spp. (compared to 0–40% of controls) and significantly reduced fungal burden, demonstrated by reduced galactomannan levels, fungal DNA, and histopathology (*p* ≤ 0.05) [19]. In a rabbit model of invasive aspergillosis, olorofim reduced serum galactomannan in a time-dependent manner (*p* < 0.001), with antifungal activity comparable to posaconazole, while remaining effective in triazole-resistant strains where posaconazole showed no benefit [49]. Similarly, in a murine coccidioidomycosis model, olorofim administered at 13.3 mg/kg three times daily improved 30-day survival and reduced fungal burden in the brain compared to fluconazole or lower/less frequent dosing of olorofim, with no evidence of rebound fungal growth [17]. Olorofim has demonstrated antagonism when combined with some azoles [50]. The proposed mechanism of antagonism is the upregulation of pyrimidine biosynthesis by azoles, which counteracts DHODH inhibition and the depletion of intracellular pyrimidine induced by olorofim [51]. In a recent phase 2 study evaluating olorofim in combination with azoles as salvage therapy for invasive fungal diseases, no pattern of reduced efficacy was observed with combination therapy, although interpretation was limited by a small number of patients for individual fungi [52].

In clinical studies, olorofim has shown promise as a salvage therapy in difficult-to-treat invasive fungal diseases caused by a range of molds and dimorphic fungi. In a phase 2b open-label, single-arm study involving 202 patients with proven or probable invasive fungal disease and limited or no therapeutic alternatives, olorofim achieved a global response rate (clinical, radiological, and mycological responses) of 29% at day 42, increasing to 75% when stable disease was included [18]. Global response rates for invasive aspergillosis were numerically comparable between patients receiving olorofim monotherapy (24/77, 31%) and those on olorofim-azole combination therapy (11/24, 46%) [52]. In contrast, among coccidioidomycosis patients, the clinical response at day 42 was numerically higher with the olorofim–azole combination (26/30, 87%) compared to olorofim alone (5/11, 46%) [52].

Olorofim demonstrated excellent tolerability and safety, with only 3% of patients discontinuing treatment due to adverse reactions, substantially lower than the observed frequency for other antifungal agents [18,53]. A phase 3 randomized clinical trial (Olorofim *Aspergillus* Infection Study [OASIS], NCT05101187) is ongoing, evaluating olorofim vs. liposomal AmB followed by standard-of-care in patients with invasive aspergillosis, with all-cause mortality at day 42 as the primary endpoint. Clinically achievable plasma concentrations (steady-state C_average_ ~3–5 mg/L) exceed the MICs for most target fungi and demonstrate excellent penetration into the lungs, brain, and other deep tissues [16,17,18]. Discrepancies between in vitro, in vivo, and clinical data highlight the need for *T. marneffei*-specific studies to advance olorofim from bench to bedside.

### 4.4. Strengths and Limitations

Our study has some limitations. First, only *T. marneffei* isolates collected from participants in Vietnam were included. Future studies should include strains across a broader geographical range in South and Southeast Asia to determine whether the individual and combined antifungal activity of AmB and olorofim hold consistent across different geographical and genetic clades. Second, we recognize that in vitro findings may not translate to real-world clinical results. Various pharmacological and clinical variables such as drug levels at the site of infection, changes in local tissue pH or protein binding, host immune response, co-infections, and the co-administration of other drugs alter the effectiveness of drugs and drug combinations in humans. Hence, our results are not definitive evidence of the absence of potential pharmacodynamic or clinical benefits. In vivo animal models or clinical studies would be the next step to investigating antifungal combination effects. Third, although previous in vitro studies evaluating olorofim activity against *T. marneffei* determined MIC visually using complete inhibition (100%) as the MIC endpoint, we used ≥95% inhibition to evaluate the antifungal activity of olorofim against *T. marneffei*, as we believe this is the most accurate and practical approximation near complete inhibition [27,28]. To our knowledge, this is the first study to assess the spectrophotometric quantification of olorofim’s growth inhibition. In our previous study, we demonstrated high inter-rater agreement (94–100%) between the visually determined CLSI MICs and alamarBlue-based MIC determination using a ≥95% inhibition threshold, supporting the reproducibility of this approach [35]. Furthermore, MICs in this study were assessed both visually and spectrophotometrically to improve endpoint reliability and facilitate a comparison with conventional CLSI methodology. Fourth, we used the checkerboard method to evaluate combination effects as a high-throughput and cost-efficient screening tool. The checkerboard assays provide a “snapshot” or static picture of fungal growth inhibition at a single time point and do not capture the time- and concentration-dependent effects of drugs, which are better assessed using the time-kill assay. We validated the findings of the checkerboard assay with those of the time-kill assay, but due to the resource intensity of time-kill assays, only one representative isolate was used for these experiments. The results from a single isolate are unlikely to fully capture inter-isolate variability in the antifungal response. Although the kinetics of olorofim may not be generalizable to the whole collection of isolates, the observed concentration-dependent antifungal effect of AmB was expected and provides an internal validation for our limited time-kill experiments. Future studies evaluating a larger number of clinical isolates are needed to confirm the reproducibility and generalizability of our findings.

Notwithstanding these limitations, our study has notable strengths. The 55 tested isolates were obtained from a prospective randomized trial of talaromycosis patients recruited from five centers across geographically diverse regions in Vietnam. These isolates are representative of globally recognized genetic clades of *T. marneffei*, with no additional or novel lineages identified in Thailand, China, northeastern India, or in travel-related cases [34]. The inclusion of both recognized genetic clades (23 northern-clade and 32 southern-clade) enhances the generalizability of our findings. We used a validated fluorescence-based alamarBlue assay to evaluate the in vitro interaction, which provides a more objective endpoint than the visual assessment of growth inhibition [35]. Finally, we implemented robust quality control measures, including internal *T. marneffei* reference isolates, two reference strains (*Candida krusei* ATCC 6258 for AmB and *Aspergillus fumigatus* for olorofim), and colony counts to confirm inoculum density.

### 4.5. Summary

Our in vitro study did not find significant interactions or synergy between AmB and olorofim, suggesting a limited benefit of this combination as induction therapy for talaromycosis. However, this does not preclude its potential clinical benefits. Our data showed consistently low MICs of olorofim against *T. marneffei* across different geographical isolates. Together with its excellent tissue penetration, long half-life, high oral bioavailability, favorable safety profile, and established effectiveness against other invasive molds and endemic fungal diseases, olorofim is a compelling candidate for consolidation and maintenance therapy in talaromycosis. We advocate for future in vivo and clinical studies to evaluate the efficacy of olorofim in this setting.

## Figures and Tables

**Table 1:** Minimum inhibitory concentrations (MICs) and combination effects of amphotericin B (AmB) and olorofim against 55 clinical *Talaromyces marneffei* isolates.

	MIC_95_ (µg/mL)		FIC_AmB_	MIC_95_ (µg/mL)		FIC_Olorofim_	FICI
	AmB	AmB + Olorofim		Olorofim	Olorofim + AmB		
Indifference; all isolates, n = 55							
Mean *	0.95	0.38	0.51	0.03	0.003	0.24	0.75
	(0.85–1.06)	(0.30–0.48)	(0.25)	(0.02–0.04)	(0.002–0.005)	(0.24)	(0.21)
Mode	1	0.5	0.5	0.03	0.001	0.5	0.56
Range	0.5–2	0.03–1	0.02–1	0.004–0.13	0.0002–0.06	0.008–1	0.52–1.03
(0.5 < FICI ≤ 1), n = 46 (84%)							
Mean *	1.02	0.36	0.43	0.03	0.004	0.26	0.70
	(0.91–1.13)	(0.28–0.47)	(0.15)	(0.02–0.04)	(0.003–0.007)	(0.21)	(0.19)
(1 < FICI ≤ 4), n = 9 (16%)							
Mean *	0.68	0.46	0.90	0.04	0.001	0.13	1.02
	(0.51–0.90)	(0.20–1.05)	(0.50)	(0.02–0.10)	(0.001–0.002)	(0.09)	(0.90)

Note: MIC represents the lowest drug concentration that achieves ≥ 95% fungal growth inhibition. * indicates geometric or arithmetic mean. Geometric means with 95% confidence intervals are reported for MICs of AmB and olorofim (alone and in combination). Arithmetic means ± standard deviations are presented for FICs of AmB and olorofim and FICIs across all 55 isolates. Abbreviations: FIC, fractional inhibitory concentration; FICI, fractional inhibitory concentration index (FIC_AmB_ + FIC_Olorofim_).

## Data Availability

All relevant data are within the manuscript and its Supporting Information Files.

## Supplementary Materials

The following supporting information can be downloaded at: https://www.mdpi.com/article/10.3390/jof12060441/s1. The supplementary files include characteristics of the clinical isolates in Supplementary Table S1: Talaromyces marneffei isolate information and Supplementary File S1: raw data from the checkerboard and time-kill assays.
