# Supplementary material for: In Vitro Evaluation of Olorofim and Amphotericin B Combination Therapy Against Talaromyces marneffei
Source: J Fungi (Basel). 2026 Jun 17;12(6):441. doi: 10.3390/jof12060441 (PMC13300783; doi:10.3390/jof12060441)
Supplement: Supplementary file 1 [file jof-12-00441-s001.zip › Supplemental Table S1.pdf]

Supplementary Table S1*: Talaromyces marneffei* **isolate information.**

***T. marneffei* isolate ID**	**Geographical origin of isolate**	**Year of isolation**	**Isolated from**	**Gender**	**CD4 count (cells/mm^3)**
11CN-03-002	Ho Chi Minh City	2012	Blood	Female	7
11CN-03-006	Ho Chi Minh City	2012	Blood	Male	17
11CN-03-007	Ho Chi Minh City	2012	Blood	Male	39
11CN-03-008	Ho Chi Minh City	2012	Blood	Female	11
11CN-03-009	Ho Chi Minh City	2012	Blood	Female	NA
11CN-03-014	Ho Chi Minh City	2012	Blood	Female	17
11CN-03-021	Ho Chi Minh City	2013	Blood	Male	29
11CN-03-029	Ho Chi Minh City	2013	Blood	Female	17
11CN-03-037	Ho Chi Minh City	2013	Blood	Female	3
11CN-03-039	Ho Chi Minh City	2013	Blood	Male	46
11CN-03-040	Ho Chi Minh City	2013	Blood	Male	18
11CN-03-048	Ho Chi Minh City	2013	Blood	Female	6
11CN-03-051	Ho Chi Minh City	2013	Blood	Male	21
11CN-03-068	Ho Chi Minh City	2013	Blood	Female	19
11CN-03-070	Ho Chi Minh City	2013	Blood	Male	23
11CN-03-078	Ho Chi Minh City	2013	Blood	Male	12
11CN-03-083	Ho Chi Minh City	2014	Blood	Male	38
11CN-03-086	Ho Chi Minh City	2014	Blood	Male	12
11CN-03-098	Ho Chi Minh City	2014	Blood	Female	3
11CN-03-104	Ho Chi Minh City	2014	Blood	Female	7
11CN-03-108	Ho Chi Minh City	2014	Blood	Male	8
11CN-03-116	Ho Chi Minh City	2014	Blood	Female	31
11CN-03-120	Ho Chi Minh City	2014	Blood	Male	34
11CN-03-121	Ho Chi Minh City	2014	Blood	Female	15
11CN-03-129	Ho Chi Minh City	2015	Blood	Female	2
11CN-03-130	Ho Chi Minh City	2015	Blood	Male	53
11CN-03-140	Ho Chi Minh City	2015	Blood	Male	4
11CN-03-147	Ho Chi Minh City	2015	Blood	Male	71
11CN-03-148	Ho Chi Minh City	2015	Blood	Female	10
11CN-03-153	Ho Chi Minh City	2015	Blood	Male	4
11CN-03-154	Ho Chi Minh City	2015	Blood	Male	3
11CN-03-158	Ho Chi Minh City	2015	Blood	Male	4
11CN-20-002	Ha Noi	2012	Blood	Male	11
11CN-20-005	Ha Noi	2012	Blood	Male	23
11CN-20-008	Ha Noi	2013	Blood	Female	3
11CN-20-016	Ha Noi	2013	Blood	Male	6
11CN-20-023	Ha Noi	2013	Blood	Female	5
11CN-20-029	Ha Noi	2013	Blood	Male	13
11CN-20-044	Ha Noi	2013	Blood	Female	3
11CN-20-065	Ha Noi	2014	Blood	Male	123
11CN-20-070	Ha Noi	2014	Blood	Male	9

11CN-20-079	Ha Noi	2014	Blood	Male	6
11CN-20-086	Ha Noi	2014	Blood	Male	8
11CN-20-091	Ha Noi	2014	Blood	Female	5
11CN-20-102	Ha Noi	2015	Blood	Male	5
11CN-20-120	Ha Noi	2015	Blood	Male	11
11CN-21-022	Ha Noi	2014	Blood	Male	9
11CN-21-031	Ha Noi	2015	Blood	Female	9
11CN-21-040	Ha Noi	2015	Blood	Female	3
11CN-21-041	Ha Noi	2015	Blood	Male	373
11CN-26-003	Quang Ninh	2013	Blood	Male	25
11CN-26-024	Quang Ninh	2015	Blood	Male	16
11CN-27-009	Hai Phong	2013	Blood	Male	13
11CN-27-012	Hai Phong	2013	Blood	Female	NA
11CN-27-017	Hai Phong	2013	Blood	Female	128

Note: Fifty-five *T. marneffei* isolates were randomly chosen from the Itraconazole *Versus* Amphotericin B for Penicilliosis (IVAP) trial. The IVAP trial recruited patients from five centers across Vietnam from 2011 to 2016.
